# Contributions of Human-Associated Archaeal Metabolites to Tumor Microenvironment and Carcinogenesis

**DOI:** 10.1128/spectrum.02367-21

**Published:** 2022-02-28

**Authors:** Mingwei Cai, Shruthi Kandalai, Xiaoyu Tang, Qingfei Zheng

**Affiliations:** a Institute of Chemical Biology, Shenzhen Bay Laboratory, Shenzhen, Guangdong, China; b Department of Radiation Oncology, College of Medicine, The Ohio State University, Columbus, Ohio, USA; c Center for Cancer Metabolism, James Comprehensive Cancer Center, The Ohio State University, Columbus, Ohio, USA; d School of Pharmaceutical Sciences, Nanjing Tech University, Nanjing, China; Nanchang University

**Keywords:** archaea, metabolites, carcinogenesis, gut, oral, contig

## Abstract

There is increasing awareness that archaea are interrelated with human diseases (including cancer). Archaea utilize unique metabolic pathways to produce a variety of metabolites that serve as a direct link to host-microbe interactions. However, knowledge on the diversity of human-associated archaea is still extremely limited, and less is known about the pathological effects of their metabolites to the tumor microenvironment and carcinogenesis. In the present study, we performed a large-scale analysis of archaea and their cancer-related metabolites across different body sites using >44,000 contigs with length >1,000 bp. Taxonomy annotation revealed that the occurrence and diversity of archaea are higher in two body sites, the gut and the oral cavity. Unlike other human-associated microbes, the nonmetric multidimensional scaling (NMDS) and permutational multivariate analysis of variance (PERMANOVA) analyses have shown no difference of archaeal compositions between Easterners and Westerners. Likewise, protein annotation suggests that genes encoding cancer-related metabolites (e.g., short-chain fatty acids and polyamines) are more prevalent and diverse in gut and oral samples. Archaea carrying these metabolites are restricted to *Euryarchaeota* and the TACK superphylum (*Thaumarchaeota*, *Aigarchaeota*, *Crenarchaeota*, and *Korarchaeota*), especially methanogenic archaea, such as *Methanobacteria*.

**IMPORTANCE** More evidence suggests that archaea are associated with human disease, including cancer. Here, we present the first framework of the diversity and distribution of human-associated archaea across human body sites, such as gut and oral cavity, using long contigs. Furthermore, we unveiled the potential archaeal metabolites linking to different lineages that might influence the tumor microenvironment and carcinogenesis. These results could open a new door to the guidance of diagnosing cancer and developing new treatment strategies.

## INTRODUCTION

Accumulating evidence reveals that microbial metabolites produced within the human body serve as an important factor, directly influencing the progression of pathological conditions such as cancer (1–4). As a double-edged sword, some microbial metabolites, such as butyrate, exhibit significant functions in the suppression of inflammation and cancer (5, 6), whereas others, such as secondary bile acids, promote carcinogenesis (7). As a consequence, investigations on metabolites of human microbiome may shed light on the elusive interplay between microbe and human. Despite the majority of microbes in humans being bacteria (8), the emerging evidence of the interactions of minor communities, such as archaea, fungi, parasite, and viruses, with human health and disease suggests an important role of archaea to human individuals (9–14).

Similar to bacteria, archaeal lineage is an important component of life in diverse ecosystems, such as the human body (8, 15). The first human-derived archaeal isolate was obtained from the gastrointestinal tract more than 40 years ago (16–19). Since then, archaea have been isolated from other body sites, including oral mucosa (20), subgingival plaque (20), and human colostrum and milk (21). Up until now, these isolates have been restricted to methanogenic and halophilic archaea, e.g., Methanobrevibacter smithii, Methanomassiliicoccus luminyensis, Methanomethylophilus alvus, and Haloferax massiliensis. Recent analyses with high-throughput sequencing amplicons reveal a more diverse archaeal community present across different human body sites (8, 22). Although there is no direct evidence linking archaea to certain human morbidities (10, 12), archaea share some characteristics with known pathogens, such as ample access to a host and capabilities to colonize and coexist with other species in a host (through components such as membrane-bound adhesin-like proteins) (12, 23). Furthermore, the abundance of halophilic and/or methanogenic archaea has proven to be positively or negatively correlated with human diseases, e.g., periodontal disease (24) and colorectal cancer (25), suggesting a potential effect of archaea on human disease states. The proportion of archaea in the microbial community can increase up to 25% in certain diseases (26). On the contrary, some archaea are beneficial to human health. For instance, the trimethylamine-degrading methanogenic archaea can serve as live biotherapeutic products (also known as “archaebiotics”) to prevent cardiovascular diseases (27). It is therefore important and meaningful to determine the underlying interactions between the archaeal component of the human microbiome and diseases, especially cancer (28).

The relative abundance of archaea is lower than their bacterial counterparts in the human body, and only a minority of archaea can be cultivated or isolated for in-depth analysis, due to their unique metabolic and physiological characteristics (15, 29). The development of high-throughput sequencing techniques in recent years has facilitated the availability and analysis of archaeal sequences. In the present study, we utilized more than 44,000 metagenome-assembled contiguous sequences (contigs) with length of >1,000 bp from publicly available databases to investigate the diversity of archaea, especially the cancer-related metabolites in different body sites. Microbial metabolites are believed to be the essential bridge connecting microbiota, tumor microenvironment, and cancer development (28, 30). Our study provides a framework for better investigating the correlation of archaeal metabolites with cancer.

## RESULTS

### Composition of archaea in/on human body.

**FIG 1:**
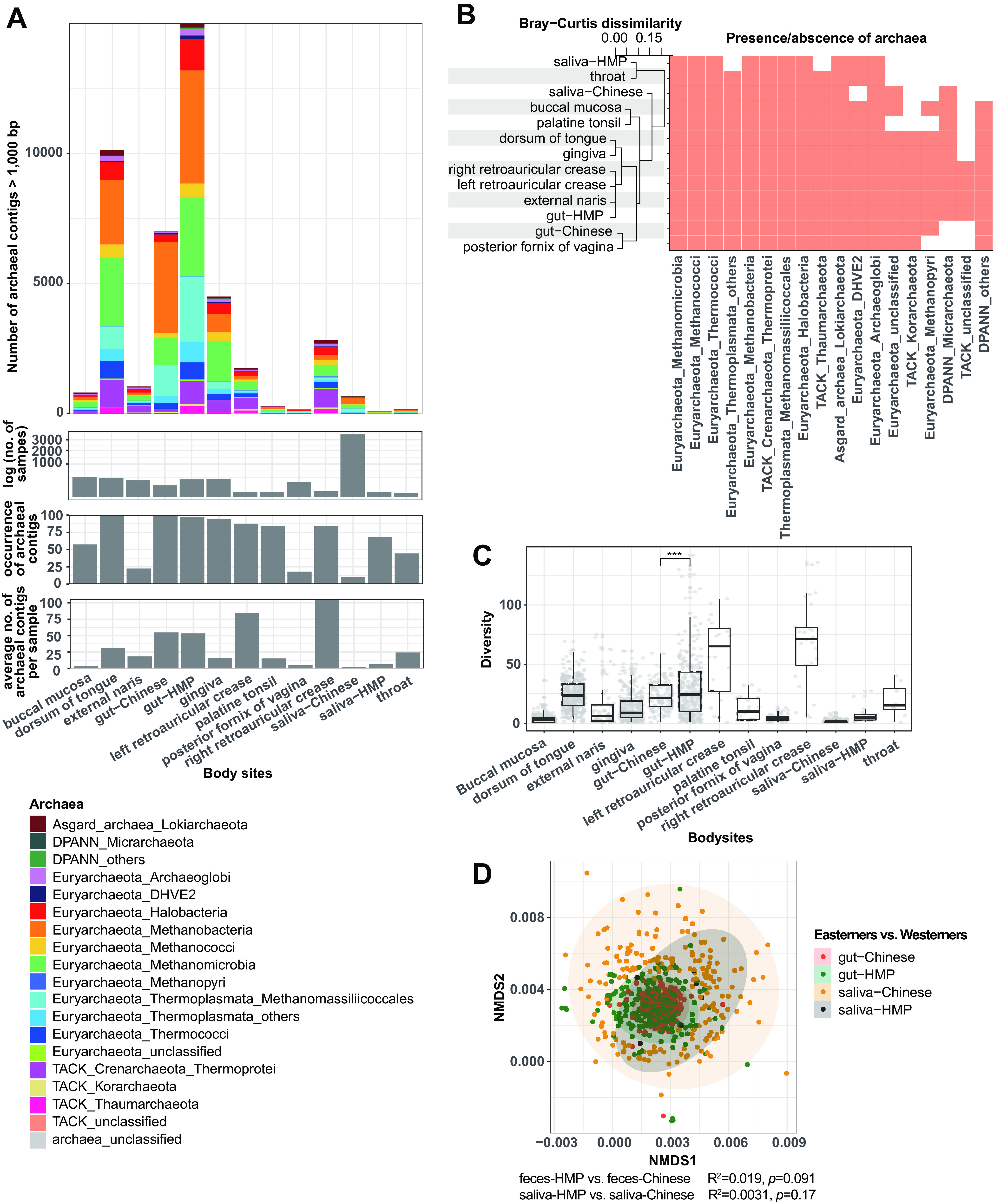
Diversity and distribution of archaea in humans. (A) Number of different archaeal contigs (>1,000 bp) in each body site. The bottom three histograms summarize the total number of samples used for analysis, occurrence of archaeal contigs, and average number of archaeal contigs per sample, respectively. (B) Presence/absence of archaea and their Bray-Curtis dissimilarity across body sites at the species level. (C) Diversity of archaea across different body sites at the species level. ***, *P* < 0.001. (D) NMDS analysis of the archaea composition at different body sites.

First, we obtained 32,451,655 contigs from 5,632 samples available in public databases (Table S1). These data sets originated from different body sites, including buccal mucosa, dorsum of tongue, external naris, gut, gingiva, hard palate, retroauricular crease, palatine tonsil, cubital fossa, saliva, throat, and vagina. After taxonomic assignment, we obtained 44,760 archaeal contigs (>1,000 bp) from 1,818 samples, with numbers ranging from 183 to 14,994 across different body sites. Specifically, the occurrence of archaeal contigs was higher in body sites like gut samples from Chinese individuals (gut-Chinese; 100%, i.e., present in 100% of the investigated samples), dorsum of tongue (99.1%), gut samples from the Human Biome Project (gut-HMP; 97.2%), and gingiva (94.4%), whereas the average number of archaeal contigs was higher in samples retrieved from right retroauricular crease (105.1), left retroauricular crease (84.3), gut-Chinese (54.9), and gut-HMP (53.4; Fig. 1A and Table S1). Although archaea from the left/right retroauricular creases were almost shared with gut-HMP and external naris at the phylum/class level with a Bray-Curtis dissimilarity of ∼0.05 (Fig. 1B), the archaeal α-diversity of samples from retroauricular creases was much higher than that of those from other body sites (*P < *0.001) at the species level (Fig. 1C).

### Similarities and differences between Easterners and Westerners.

Important factors influencing the presence and diversity of microorganisms include geography and ethnicity (related to aspects such as host genetics, life history, and diet) (31, 32). Hence, we compared the diversity and similarity of archaeal communities from gut and saliva samples of Easterners and Westerners. We observed that samples from Westerners (gut-HMP) had an archaeal diversity higher than that of samples from Easterners (gut-Chinese; *P < *0.001), whereas no difference in archaeal diversity was observed between the saliva-HMP and saliva-Chinese (Fig. 1C). Further, analyses of archaeal composition using nonmetric multidimensional scaling (NMDS) and permutational multivariate analysis of variance (PERMANOVA) showed no difference for fecal (R^2^ = 0.019, *P = *0.091) and saliva (R^2^ = 0.0031, *P = *0.17) samples between Easterners and Westerners at the species level (Fig. 1D).

### Archaea and their metabolites related to carcinogenesis.

**FIG 2:**
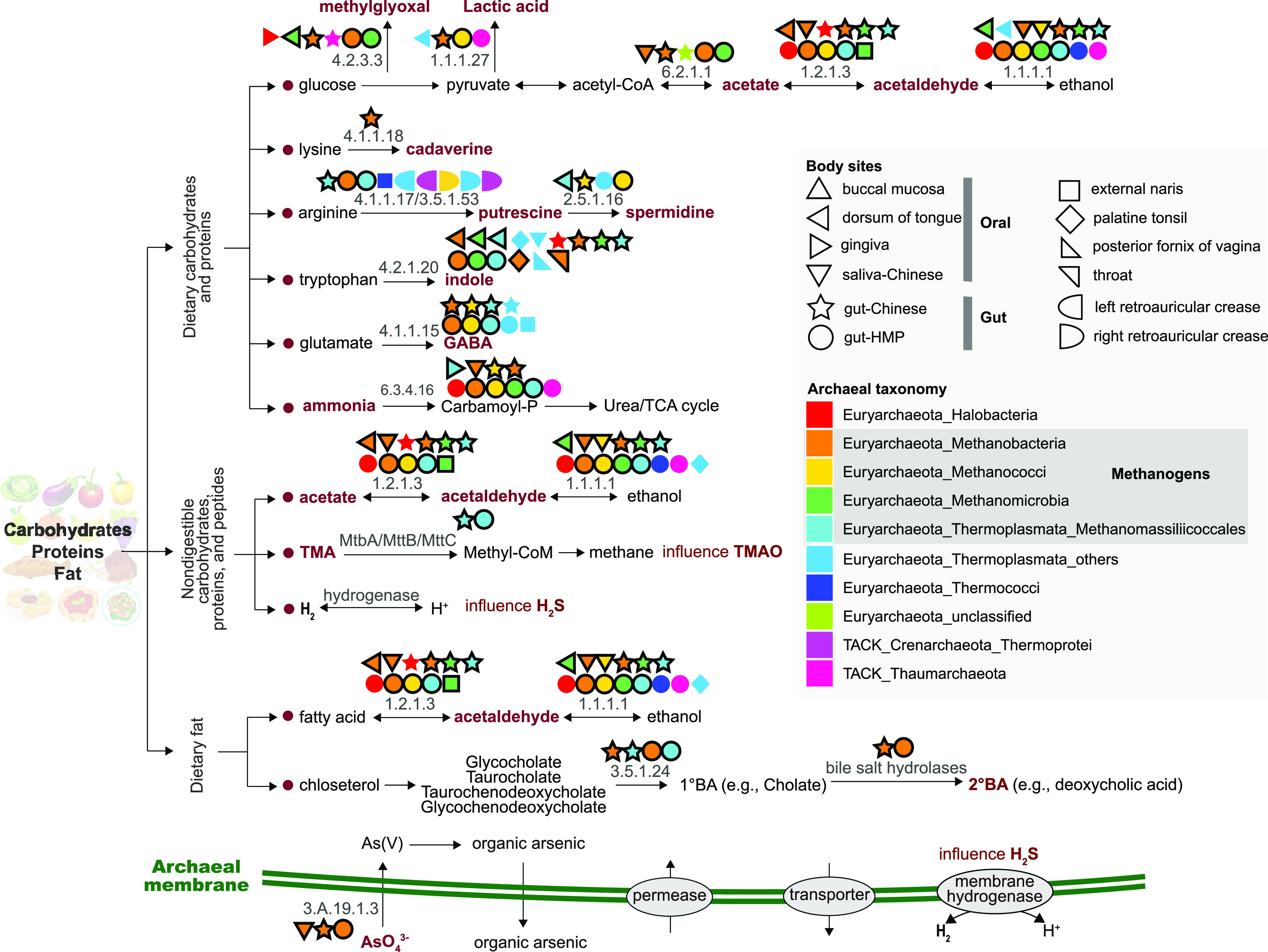
Archaeal metabolites that are directly or indirectly involved in the initiation and/or progression of carcinogenesis. The pertinent enzymes of the metabolites are also shown in the figure. Symbol shape and color represent different body sites and archaeal taxa, respectively. Symbols with black borders denote methanogens. GABA, γ-aminobutyric acid; TMA, trimethylamine; TMAO, trimethylamine N-oxide; 1°BA, primary bile acid; 2°BA, secondary bile acid. Descriptions of enzymes are available in Table S3. Detailed information of the presence of genes and those encoding hydrogenases and TCDB is available in Fig. S1 and S2 and Table S4, respectively.

To determine the potential connections of archaeal metabolites to carcinogenesis, the archaeal contigs were processed to annotate the functional genes with numerous databases and software (see Materials and Methods). We found several types of archaeal metabolites potentially linked to carcinogenesis, including short-chain fatty acids (SCFA; i.e., acetate and lactic acid) (3, 33), indoles (3, 33), polyamines (i.e., cadaverine, putrescine, and spermidine) (3, 33), ammonia (33), secondary bile acids (2°BA) (3, 33), methylglyoxal (34–38), acetaldehyde (3), γ-aminobutyric acid (GABA) (39), and arsenic (40), as well as other metabolites, including H_2_ and trimethylamine (TMA), that could influence the production of carcinogens H_2_S (3) and trimethylamine N-oxide (TMAO) (41) (Fig. 2 and Fig. S1 and S2). The known effects of these oncogenic metabolites on the host include influencing microbiota modulation, inflammation, energy supply, and reactive oxygen species (ROS) production, as well as affecting host signaling pathways through modification of histones or essential enzymes in metabolic pathways (Table 1). Although identified in diverse body sites, genes encoding these metabolites, like methylglyoxal and acetaldehyde, were more prevalent and diverse in oral (buccal mucosa, dorsum of tongue, and saliva-Chinese) and gut (gut-Chinese and gut-HMP) samples (Fig. 2), representing 64.7% and 100% of the investigated metabolites, respectively. On the other hand, archaea in other body sites, such as retroauricular creases, throat, and vagina, were more likely to produce polyamines (Fig. 2).

**TABLE 1:** Summary of major archaeal metabolites and their associations with carcinogenesis

Archaeal metabolites	Known effects on host^*a*^
Acetate	Anti-inflammation, tumor proliferation, intestinal barrier function
Lactic acid	ROS production, intestinal barrier function
Polyamines (cadaverine, putrescine, and spermidine)	Inflammation, ROS production, genotoxicity, DNA repair/protection
Indole	DNA damage, anti-inflammatory
Acetaldehyde	Inflammation, DNA damage, aberrant signaling pathways
Methylglyoxal	Aberrant signaling pathways
GABA	Aberrant signaling pathways
Ammonia	ROS production, genotoxicity, tumor proliferation
TMA/TMAO	Inflammation, aberrant signaling pathways
2°BA	Microbiota modulation, cellular differentiation, apoptosis, ROS production, genotoxicity
H_2_/H_2_S	DNA damage, inflammation, ROS production, genotoxicity
Inorganic arsenic	DNA damage, ROS production, genotoxicity

a The known effects are reported elsewhere (3, 33–41).

To verify whether the abundance of genes encoding these metabolites is different between healthy individuals and cancer patients, we selected a representative data set for comparison. We uncovered that the abundance of genes encoding enzymes (EC 6.2.3.3, EC 6.2.1.1, EC 1.1.1.1, EC 4.1.1.18, EC 4.2.1.20, EC 4.1.1.15, bile acid hydrolase, and 3.A.19.1.3) for cancer-related metabolites was much higher (*P < *0.05) in colorectal cancer patients than in healthy individuals (Fig. S3), suggesting the potential of these archaea-derived metabolites to carcinogenesis.

Archaea potentially producing these metabolites were classified into 10 lineages within the *Euryarchaeota* and the TACK superphylum (Fig. 2). Although other archaeal lineages, such as *Lokiarchaeota* (Asgard archaea) and *Micrarchaeota* (DPANN), were identified in human samples (Fig. 1), they were not discussed in the next-step analyses because these contigs encode mainly housekeeping genes (Table S2). Additionally, the proportion of these species is relatively low in human samples and can seldomly be retrieved through metagenomic amplification. Here, methanogens, including *Methanobacteria*, *Methanococci*, *Methanomicrobia*, and *Methanomassiliicoccales*, were more frequently associated with the production or consumption of these metabolites, and they may be exclusively responsible for the transformation of several metabolites (such as TMA and 2°BA) in the human body. Specifically, *Methanobacteria* were the most popular methanogen and carried genes for all of the investigated metabolites. In addition, the presence of metabolites produced by other archaeal lineages (such as *Halobacteria*, *Thermococci*, *Thermoprotei*, and *Thaumarchaeota*) may also be responsible for the cycling of metabolites related to carcinogenesis (Fig. 2 and Table 1). Further, cooccurrence analysis showed a positive correlation between methanogenic and halophilic archaea and the known cancer-related pathogen Helicobacter pylori (28) (Fig. S4).

In line with these metabolites, we identified the correlated membrane-bound transporters, such as the polyamine exports (PF00324 and PF07690) and 2°BA transporters (PF01758; Fig. S1B and Table S4). Genes encoding these transporters are diversely distributed across different body sites, ensuring that the archaeal metabolites can be transferred into the host cells or to their mutualistic partners, such as bacteria and fungi, and can further influence the host pathophysiological states directly and/or indirectly.

## DISCUSSION

Microbial communities interact with human cells through the production and regulation of metabolites to maintain cellular metabolism and the immune and neuronal systems (42, 43). In the past decade, related investigations have highlighted the increasing risk of dysbiosis of the archaeal or bacterial microbiome associated with diseases such as colorectal cancer (25) and breast cancer (2). Archaea (specifically methanogenic archaea) have been indicated to be essential components and represent keystone species in metabolic processes in the human body (22, 44, 45). Although recent studies have revealed their correlations with diseases (11, 12, 25, 29), more research regarding different archaea and their potential pathological roles in carcinogenesis is urgently needed.

The interactions of archaea with cancer can be traced back to as early as the 1980s, when it was unveiled that Africans with higher levels of methanogenic archaea showed a lower risk for large bowel cancer (31). Lately, shotgun metagenomic analyses have indicated that other than the methanogenic archaea, the abundance of halophilic archaea was positively correlated with colorectal cancer (25), underlying their potential impact to humans. Although archaea such as *Thaumarchaeota* and other unclassified *Euryarchaeota* were also identified in or on human body (8, 22), to the best of our knowledge, their potentials with cancer have not been reported in depth. For the first time, we have identified genes encoding cancer-related metabolites affiliated with nonmethanogenic or nonhalophilic archaea, such as *Thermococci*, *Thermoprotei*, and *Thaumarchaeota*, at the contig level (Fig. 2). The *Thaumarchaeota* in the gut may participate in biochemical processes, such as the oxidation of candidate carcinogen ammonia (46, 47) as supplied from the deaminated proteinaceous material, while *Thermococci* and *Thermoprotei* may be more responsible for polyamine production (48). Recent reports using 16S rRNA genes have also revealed the presence of these archaea in the gastrointestinal tract, lung, and skin (25). These facts support our observation in this study. However, further molecular experiments are still needed to delineate the participation of these archaea in cancer progression.

The gut, oral cavity, skin, and vagina harbor a peculiar set of highly diverse microbial consortia, with diversity highest in gut and oral cavity (49–52). This phenomenon is also applicable to archaea, as we identified an archaeal diversity in oral and gut samples (gut, dorsum of tongue, and gingiva) higher than that in other body sites (Fig. 1). Similarly, genes encoding cancer-related metabolites have been detected in the oral cavity and gut in most cases, but they were seldomly identified in other body sites (Fig. 2). The decrease of beneficial metabolites or the increase of detrimental metabolites to an alarming level has been substantiated to be associated with cancer in the oral cavity and gut (49, 53). Thus, the equilibrium of archaea-related metabolites in the oral cavity and/or gut, especially the archaeon-specific TMA, may be pivotal to human health.

**FIG 3:**
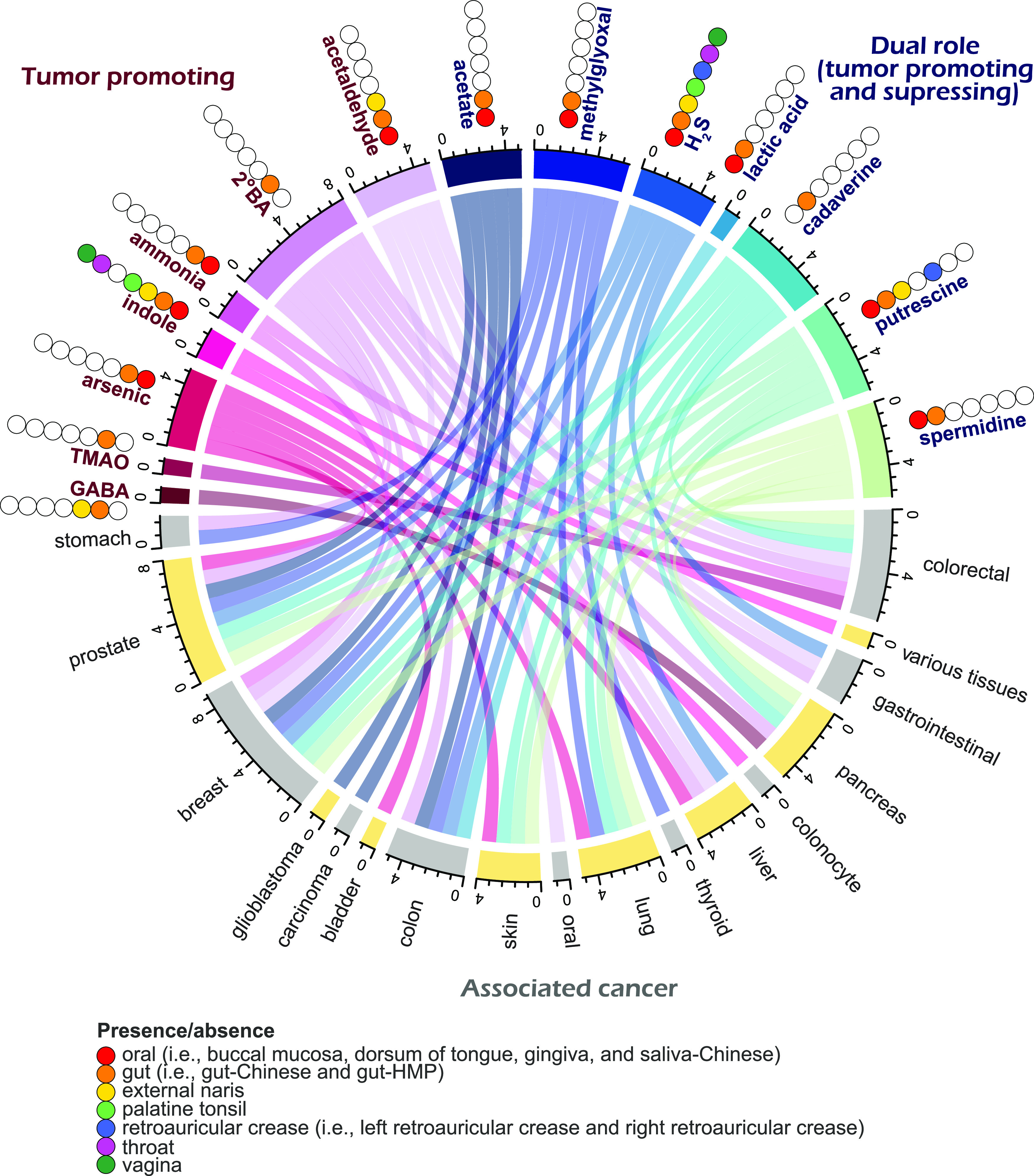
Archaeal metabolites and their potential roles with various cancers. Relations were organized and displayed on the basis of previous reports (7, 40–42, 61, 87–89).

The dysbiosis of gut and oral microbiome is linked to other diseases of different systems, including diabetes (54), pneumonia (55), and cardiovascular disease (56). Meanwhile, recent gut-brain axis studies also observed a significant link between the gut microbiome and some complex diseases, such as Alzheimer’s disease (57) and Parkinson’s disease (58). In the present study, our literature search of these archaeal metabolites potentially links them to different non-oral or non-gut cancers, beneficially or detrimentally (Fig. 3). Carcinogens, including acetaldehyde and methylglyoxal, have been reported to influence human health by regulating protein modifications or perturbing protein/chromatin structure (59, 60). Thus, the produced metabolites in gut and oral cavity might also increase the cancer risk in other organs, such as liver and breast (61). Since archaea are difficult to enrich or isolate, these correlations could be further analyzed using a combination of metagenomics and metatranscriptomics on a larger scale.

Bacterial biomarkers like *Fusobacterium* species (62), Porphyromonas asaccharolytica, and Peptostreptococcus stomatis (63) hold promise for early diagnosis of a certain cancer. Similar studies have been performed for archaea, showing that several representatives of methanogenic and halophilic archaea may be used as biomarkers for colorectal tumor development (25). In the present study, screening of contigs has shown that cancer-related archaeal biomarkers are also present in samples from healthy individuals (Fig. S5). A possible reason for this inconsistency is that the taxonomic annotation in the report was assigned using short reads (25), which might not be accurate for taxonomy assignment to the species level. Studies using functional genes have been applied for predictions of diseases, such as dental caries (53) and colorectal cancer (64). Indeed, the application of functional genes as biomarkers is more reasonable, as (i) the human microbiota is fluctuating because factors such as geographic location, diet (31, 32), and age (65) influence the presence, abundance, and diversity of archaea in humans and (ii) archaea may have functional redundancy (i.e., duplicate genes across archaea that maintain biochemical functions over time, independent of variance in taxonomic composition), as reported for microbes in other ecosystems (66). Thus, the identification of archaeal marker genes for cancer, especially those with high-level transcription increases in patients, is needed for early diagnosis of archaea-induced cancer.

The human microbiome is a complex aggregate of the microbes. Usually, microbes like bacteria, archaea, and fungi coexist to interact with their host through an elaborate network (8, 67). In human body, methanogenic archaea have been reported to be positively correlated with bacteria (68), and their mutualism has also been shown in humans and a humanized gnotobiotic mouse model (69). Further, methanogens are capable of syntrophic interactions with bacteria to enhance the production of SCFA (29), which play a role in cancer suppression within the host (33). However, the dysbiosis of microbes could interrupt the mutualism between bacteria and methanogenic archaea and lead to diseases like colorectal cancer (25). As no proof for such archaeal pathogens exists (10, 12), these human-associated archaea are more likely to function in cooperation with other microbes, such as the known cancer-related pathogen Helicobacter pylori (Fig. S4).

In summary, we performed a large-scale analysis of human-associated archaea and cancer-related metabolites using more than 44,000 contigs across different body sites. Results revealed that although archaea were identified in all body sites, the occurrence and diversity were higher in samples from the oral cavity and gut, with archaeal composition being similar between Easterners and Westerners. Similarly, genes encoding cancer-related metabolites were more diverse and prevalent in oral and gut samples, and these metabolites were affiliated with *Euryarchaeota* and the TACK superphylum, especially methanogenic archaea. These findings largely improved our understanding of the connections between archaea and human tumor microenvironment, thereby shedding light on early diagnosis and therapeutic design for cancer. However, similar to bacteria, these associations need to be further experimentally verified before instruction or application in therapy.

## MATERIALS AND METHODS

### Data set acquisition.

Contigs for analysis were retrieved from four different data sets: human saliva samples of Chinese individuals (saliva-Chinese) (70), human fecal samples of Chinese individuals (gut-Chinese) (64), human saliva samples from European individuals (71), and samples from the Human Microbiome Project (HMP) that included body sites like buccal mucosa, dorsum of tongue, throat, and vagina (72). We acquired a total of 5,614 samples (3,591 for saliva-Chinese, 128 for gut-Chinese, and 1,859 for HMP), and these samples were retrieved from healthy Eastern and Western subjects (Table S1). Contigs for the samples were downloaded directly from NCBI (https://www.ncbi.nlm.nih.gov/) based on the published BioProject accession numbers or from the HMP website (https://portal.hmpdacc.org/) with specific filters “HMP” and “Wgs Assembled Seq Set.” It should be noted that the publicly available data sets were from different studies and, thus, the differences in sampling, sequencing, and/or contig binning methods may have potential effect on our results.

### Contig taxonomic annotation.

To increase taxonomy assignment accuracy, the downloaded contigs were first filtered using a custom script to remove sequence with length of <1,000 bp. Kaiju (v1.7.3) and Kraken2 (v2.0.8-beta) were both introduced to perform taxonomy annotation with the default parameters against the default databases (e.g., NCBI nonredundant database) (73, 74). The Kaiju software was used because it contained a detailed database for archaea classification, and the Kraken2 software was supplemented for validation. Finally, we had a total of 44,760 archaeal contigs from 1,818 samples (Table S1).

### Archaeal protein annotation.

Open reading frames (ORFs) of archaeal contigs were called using the “-p meta” option in Prodigal (v2.6.3) with default parameters (75). For function annotation, all proteins were annotated using the standalone software eggNOG-mapper (v2) (76, 77) and InterProScan (v5.42-78.0) (78). Archaeal clusters of orthologous genes (arCOGs) downloaded in May 2020 were used for archaeal-specific protein annotation using DIAMOND (v0.9.24) (79) with an E value of 1E-10. The annotated putative [NiFe] and [FeFe] hydrogenases sequences were compared against the HydDB database (80) for subgroup classification using DIAMOND with an E value of 1E-10. Metabolite transporters were annotated using the transporter classification database (TCDB) downloaded in May 2020 using DIAMOND with an E value of 1E-10 (81).

### Relative abundance of cancer-related genes.

Raw metagenomic reads for colorectal cancer patients (*n* = 20) and healthy individuals (*n* = 28) were downloaded from BioProject PRJEB10878 (64). Raw reads were quality-controlled using the sickle (v1.33) with the option “-q 25,” and the human-origin reads were removed using bbmap (v35.85) against the HG19 genome. The abundance of raw reads was processed using the software bwa (v0.7.17), samtools (v1.10), and bbmap (v35.85) with the default setting against the curated archaeal cancer-related genes. Finally, the mapped reads were normalized to per million reads with a custom script. The group comparison was performed with the general linear hypothesis test and Tukey procedures embedded in “multcomp” package.

### Cooccurrence of human-associated archaea with pathogens.

A set of 20 publicly available gut metagenomics from colorectal cancer patients were downloaded from the European Nucleotide Archive under the project number PRJEB7774 (Table S5). Raw reads were trimmed using Trimmomatic (v0.39) (82) with the parameter “-phred33 MINLEN:60.” The qualified reads were taxonomically assigned using the Kaiju (v1.7.3) as mentioned above.

To evaluate the cooccurrence patterns of the archaea with pathogens, reads were first tested using the checkerboard score (C-score) through the function “oecosimu” with the parameters “matrix, nestedchecker, method = “swap”, nsimul = 10000,” which also calculated the standardized effect size (SES) to avid biases of raw C-score value. The C-score of 939 and SES score of 1.59 imply that microorganisms in gut are distributed nonrandomly (83). Then, the cooccurrence network analysis was performed at the genus/species level based on the previous method (83, 84). Only correlations with a Spearman’s ρ of >0.6 and Benjamini–Hochberg adjusted *P* value of <0.01 were kept for analyses. The resulted network was imported into Gephi (version 0.9.2) (85), and the cooccurrence patterns were visualized through calculation with Fruchterman-Reingold algorithm.

### Statistical analysis.

Statistical analysis and visualization were performed in the R software (v3.6.3) using the packages “ggplot2,” “vegan,” and “picante,” unless otherwise indicated. Bray-Curtis dissimilarity for archaeal contigs in different body sites was calculated using the package “picante.” Diversity of archaea across different body sites was analyzed at the species level. Multiple comparisons of the presence and absence of archaeal contigs among samples were performed with the general linear hypothesis test and Tukey procedures embedded in “multcomp” package. NMDS analysis of the archaea assembly in different samples at the species level was performed based on Bray-Curtis dissimilarity using the “metaMDS” function in the “vegan” package. The confidential interval for the underlying body site specific ellipse is 0.95. To determine the similarities and differences between samples from Easterners and Westerners, PERMANOVA analysis was performed using the “adonis” function in the “vegan” package with 999 permutations.

### Data availability.

The contig data sets that support the findings of this study are available in European Nucleotide Archive under BioProject PRJEB10878 (64), the National Genomics Data Centre with the accession number PRJCA003731 (70), and the HMPDACC Data Portal (https://hmpdacc.org/) (72). The data set for metagenomics is available in European Nucleotide Archive with the accession numbers listed in Table S5 under BioProject PRJEB7774 (86).
