# Supplementary material for: Contributions of Human-Associated Archaeal Metabolites to Tumor Microenvironment and Carcinogenesis
Source: Microbiol Spectr. 2022 Feb 28;10(2):e02367-21. doi: 10.1128/spectrum.02367-21 (PMC9045267; doi:10.1128/spectrum.02367-21)
Supplement: SUPPLEMENTAL FILE 2 — Supplemental material. Download Spectrum02367-21_supplemental_file2.pdf, PDF file, 1.1 MB [file spectrum02367-21_supplemental_file2.pdf]

Supplementary Figures and Tables

TITLE: Contributions of Human-associated Archaeal Metabolites to Tumor Microenvironment and Carcinogenesis

AUTHORS: Mingwei Cai[1*], Shruthi Kandalai[2,3], Xiaoyu Tang[1,4*], and Qingfei Zheng[2,3*]

AFFILIATION: [1]Institute of Chemical Biology, Shenzhen Bay Laboratory, Shenzhen, China.

[2]Department of Radiation Oncology, College of Medicine, The Ohio State University, Columbus, USA.

[3]Center for Cancer Metabolism, James Comprehensive Cancer Center, The Ohio State University

[4]School of Pharmaceutical Sciences, Nanjing Tech University, Nanjing, China

[*]**CORRESPONDENT:** Qingfei Zheng, Tzagournis Medical Research Facility, 420 W. 12th Ave Columbus, Ohio 43210, United States; E-mail: Qingfei.Zheng@osumc.edu

Xiaoyu Tang, Room B309, Gaoke International Innovation Center, Shenzhen Bay Laboratory, Shenzhen, China; E-mail: xtang@szbl.ac.cn.

Mingwei Cai, Room B322, Gaoke International Innovation Center, Shenzhen Bay Laboratory, Shenzhen, China; E-mail: caimw@szbl.ac.cn.

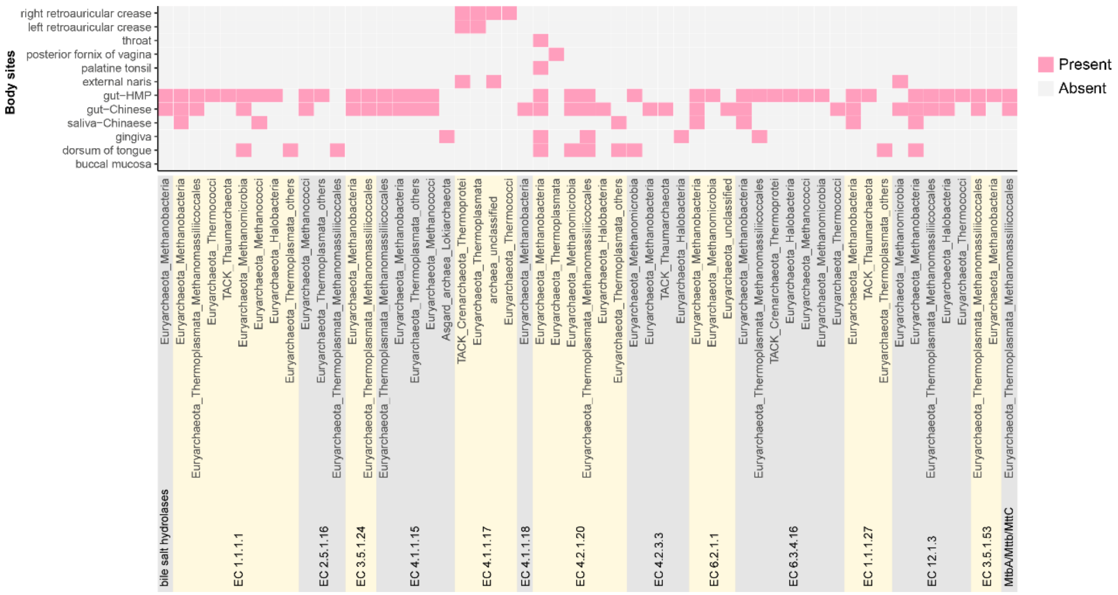

Fig. S1 Presence/absence of enzymes for cancer-related metabolites across different body sites.

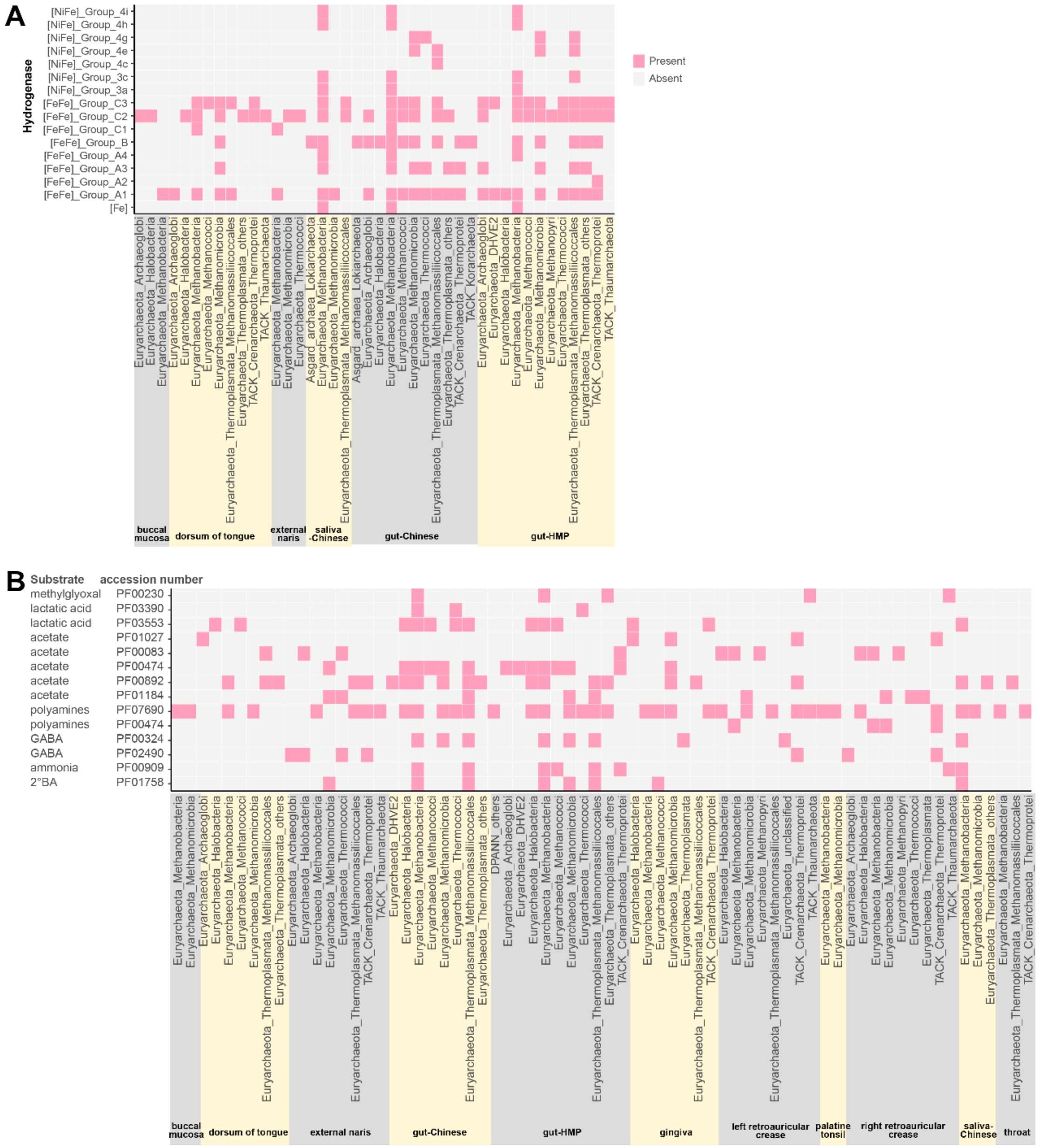

Fig. S2 (A) Presence/absence of hydrogenase for H_2 uptake or evolving. (B) Enzymes encoding transporters related to the archaeal metabolites. Detailed information is available in Table S3.

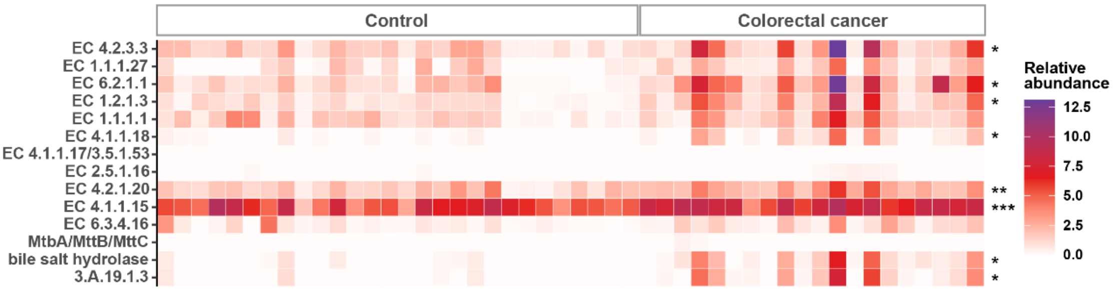

Fig. S3 Relative abundance of genes encoding enzymes in gut samples of healthy individuals (control) and colorectal cancer patients. Relative abundance denotes the read numbers per million reads. ***, $p < 0.001$; **, $p < 0.01$; *, $p < 0.05$.

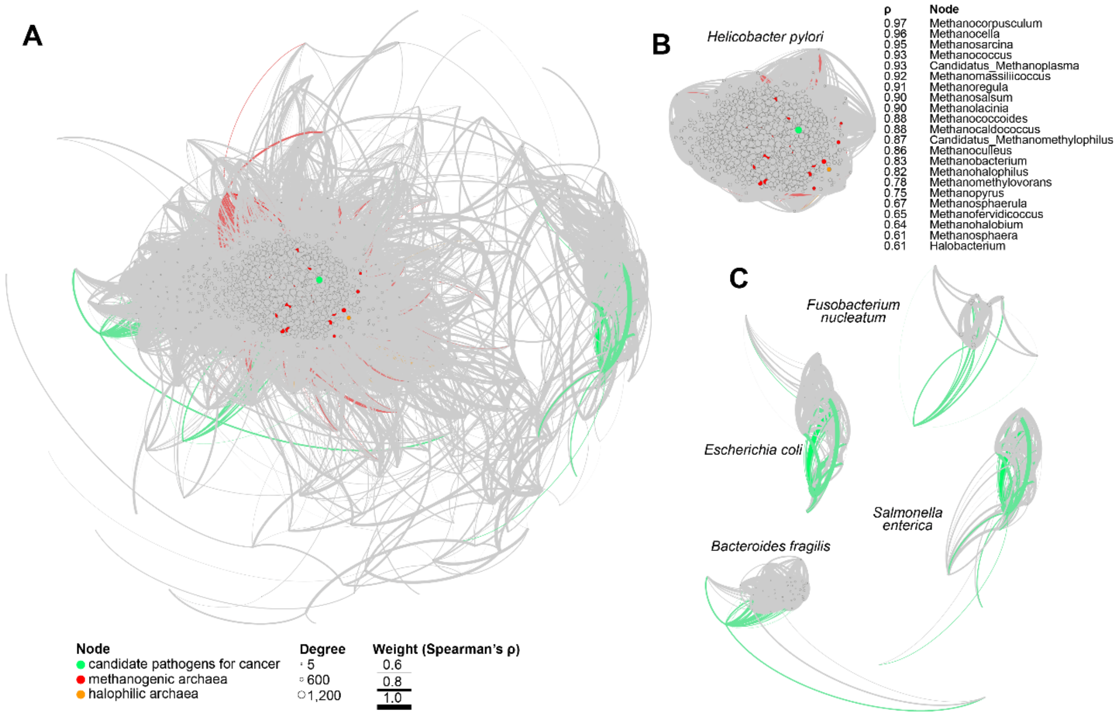

Fig. S4 Co-occurrence of archaea with cancer pathogens. (A) Overview of the co-occurrence patterns. Only positive correlations are shown. Five candidate pathogens i.e., *Helicobacter pylori*, *Fusobacterium nucleatum*, *Escherichia coli*, *Bacteroides fragilis*, and *Salmonella enterica* are marked green. (B) Direct connections of *Helicobacter pylori* with methanogenic and halophilic archaea. (C) Direct connections of *Fusobacterium nucleatum*, *Escherichia coli*, *Bacteroides fragilis*, and *Salmonella enterica* with other microorganisms.

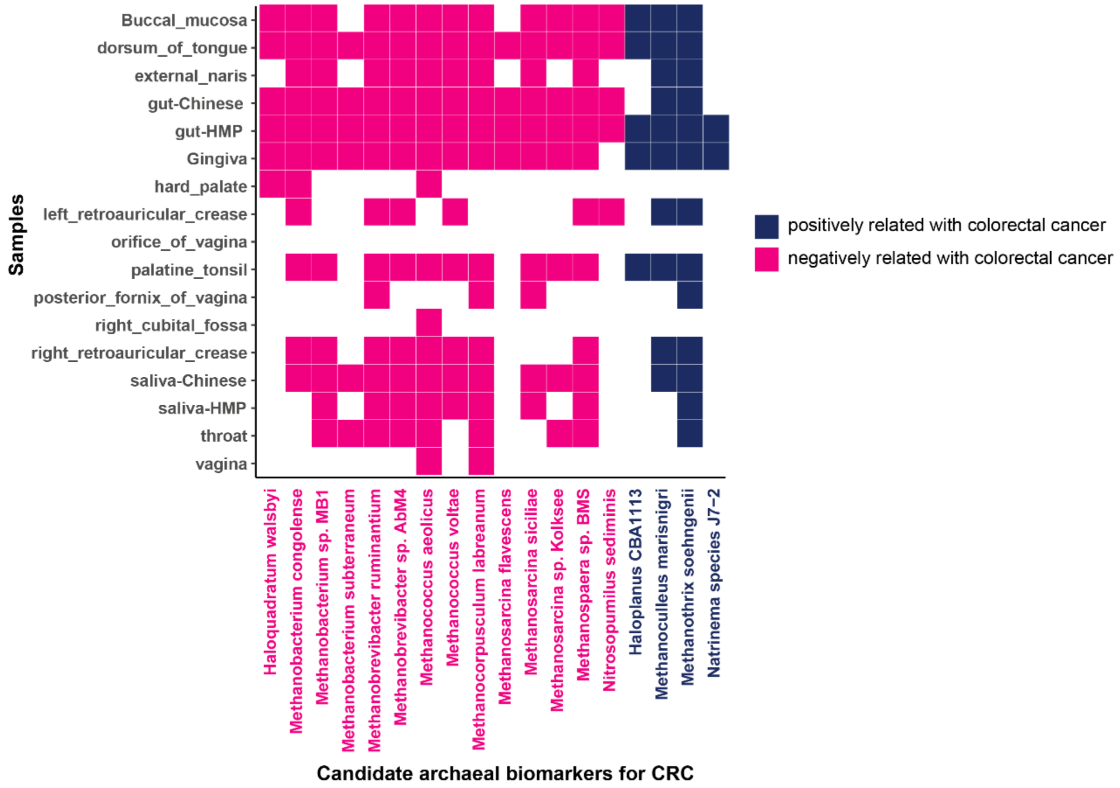

Fig. S5 Candidate archaeal biomarkers for colorectal cancer in the investigated contigs across body sites. The archaeal biomarkers were selected based on a previous study (1).

Table S1 Statistic information of the datasets used for analysis

bodysites	Easterners or Westerners	no. of samples	no. of contigs >1,000bp	no. of samples with candidate archaeal contigs	occurrence of archaeal contigs	no. of candidate archaeal contigs	average no. of archaeal contigs per sample
buccal mucosa	Westerners	376	2,476,324	216	57.4	830	3.8
dorsum of tongue	Westerners	331	10,100,586	328	**99.1**	10,127	30.9
external naris	Westerners	257	373,941	58	22.6	1,064	18.3
gut-Chinese	Easterners	128	4,458,712	128	**100.0**	7,027	**54.9**
gut-HMP	Westerners	289	6,810,956	281	**97.2**	14,994	**53.4**
gingiva	Westerners	302	6,028,963	285	**94.4**	4,502	15.8
left retroauricular crease	Westerners	24	109,232	21	87.5	1,771	**84.3**
palatine tonsil	Westerners	25	347,370	21	84.0	320	15.2
posterior fornix of vagina	Westerners	206	198,273	37	18.0	183	4.9
right retroauricular crease	Westerners	32	165,985	27	84.4	2,838	**105.1**
saliva-Chinese	Easterners	3,591	903,815	382	10.6	682	1.8
saliva-HMP	Westerners	22	212,997	16	72.7	190	6.2
throat	Westerners	18	185,050	8	44.4	194	24.3

Table S3 Description of enzymes in Fig. 2A

Enzymes	Description
3.A.19.1.3	arsenical pump membrane protein
EC 1.1.1.1	alcohol dehydrogenase, class IV
EC 1.1.1.27	lactate utilization protein
EC 1.2.1.3	actaldehyde dehydrogenase, Succinate semialdehyde dehydrogenase, a-ketoglutarate semialdehyde dehydrogenase or other NAD-dependent aldehyde dehydrogenase
EC 2.5.1.16	spermidine synthase
EC 3.5.1.24	choloylglycine hydrolase
EC 4.1.1.15	glutamate dehydrogenas
EC 4.1.1.17/3.5.1.53	ornithine decarboxylase/N-carbamoylputrescine amidase
EC 4.1.1.18	possible lysine decarboxylase
EC 4.2.1.20	tryptophan synthase, alpha chain
EC 4.2.3.3	methylglyoxal synthase/Methylglyoxal synthase-like domain
EC 6.2.1.1	acyl-CoA synthetase
EC 6.3.4.16	carbamoylphosphate synthase large/small subunit
MtbA, MttB, MttC	trimethylamine corrinoid protein

Table S4 Descriptions of TCDB for archaeal metabolites transport

**Substrate**	**TCDB**	**accession number in PF database**	**Annotation#**
methylglyoxal	1.A.8.9.3	PF00230	major aquaglyceroporin, LmAQP1: transports water, glycerol, methylglyoxal, trivalent metalloids such as arsenite and antimonite, dihydroxyacetone and sugar alcohols
lactic acid	2.A.24.2.2	PF03390	malate:lactate antiporter (substrates include: S-lactate, R-lactate, S-malate and S-citramalate)
lactic acid	2.A.35.1.2	PF03553	malate + H+: lactate + Na+ antiporter, MleN
acetate	1.A.14.2.2	PF01027	the YbhL (AceP) protein. Possibly a pmf-dependent acetate uptake transporter.
acetate	2.A.1.6.8	PF00083	the acetate/monochloroacetate (haloacid) permease
acetate	2.A.21.5.3, 2.A.21.7.2	PF00474	Na+-dependent short chain fatty acid transporter SLC5A8 (tumor suppressor gene product, down-regulated in colon cancer) (substrates: lactate, pyruvate, acetate, propionate, butyrate (Km ≈1 mM))
acetate	2.A.7.3.1	PF00892	putative acetate efflux pump, MadN
acetate	2.A.96.1.1, 2.A.96.1.3, 2.A.96.1.3	PF01184	acetate/succinate transporter, SatP or YaaH of 196 aas and 6 TMSs (Sá-Pessoa et al. 2013). It is specific for acetate (a monocarboxylate) and for succinate (a dicarboxylate), with affinity constants at pH 6.0 of 1.24 ± 0.13 mM for acetate and 1.18 ± 0.10 mM for succinate
polyamines	2.A.1.2.16, 2.A.1.2.43, 2.A.1.2.64, 2.A.1.2.66, 2.A.1.2.67	PF07690	polyamines (spermine, spermidine, putrescine); paraquat; methylglyoxal bis(guanylhydrazone):H+ antiporter (in the plasma membrane) (activated by phosphorylation); The multidrug efflux pump, Qdr3 (exports polyamines, quinidine, barban, cisplatin and bleomycin)
polyamines	2.A.3.4.5	PF00324	the polyamine (putrescine > spermidine > spermine) exporter, Tpo5p (Ykl174c) [found in the Golgi or post-Golgi secretory vesicles; induction:spermine > spermidine > putrescine] (Igarashi and Kashiwagi 2010).
GABA	2.A.18.5.1	PF02490	vesicular γ-aminobutyric acid (GABA) and glycine transporter
GABA	2.A.22.3.2, 2.A.22.3.6	PF00209	γ-Aminobutyric acid (GABA):Na+:Cl- symporter
ammonia	1.A.11.1.1, 1.A.11.1.2, 1.A.11.1.3, 1.A.11.1.4, 1.A.11.1.5, 1.A.11.1.6, 1.A.11.2.1, 1.A.11.2.2,	PF00909	ammonia transporter and regulatory sensor

	1.A.11.2.3, 1.A.11.2.4, 1.A.11.3.1, 1.A.11.3.2, 1.A.11.3.3, 1.A.11.3.4		
2°BA	2.A.28.1.2, 2.A.28.1.5, 2.A.28.1.6, 2.A.28.1.7, 2.A.28.1.9	PF01758	liver/ileal bile acid:Na+ symporter

[#]Detailed descriptions of these functions are available in the TCDB website https://www.tcdb.org/search/index.php?query=&type=system.

Table S5 List of metagenomic datasets for analysis

Sample Name	Cancer type	Project
SAMEA3136748	Colorectal cancer	PRJEB7774
SAMEA3136769	Colorectal cancer	PRJEB7774
SAMEA3136751	Colorectal cancer	PRJEB7774
SAMEA3136738	Colorectal cancer	PRJEB7774
SAMEA3136747	Colorectal cancer	PRJEB7774
SAMEA3136743	Colorectal cancer	PRJEB7774
SAMEA3136754	Colorectal cancer	PRJEB7774
SAMEA3136765	Colorectal cancer	PRJEB7774
SAMEA3136724	Colorectal cancer	PRJEB7774
SAMEA3136755	Colorectal cancer	PRJEB7774
SAMEA3136766	Colorectal cancer	PRJEB7774
SAMEA3136728	Colorectal cancer	PRJEB7774
SAMEA3136756	Colorectal cancer	PRJEB7774
SAMEA3136753	Colorectal cancer	PRJEB7774
SAMEA3136737	Colorectal cancer	PRJEB7774
SAMEA3136726	Colorectal cancer	PRJEB7774
SAMEA3136734	Colorectal cancer	PRJEB7774
SAMEA3136758	Colorectal cancer	PRJEB7774
SAMEA3136750	Colorectal cancer	PRJEB7774
SAMEA3136742	Colorectal cancer	PRJEB7774
SAMEA3541516	Control	PRJEB10878
SAMEA3541517	Control	PRJEB10878
SAMEA3541518	Control	PRJEB10878
SAMEA3541519	Control	PRJEB10878
SAMEA3541520	Control	PRJEB10878
SAMEA3541521	Control	PRJEB10878
SAMEA3541522	Control	PRJEB10878
SAMEA3541523	Control	PRJEB10878
SAMEA3541524	Control	PRJEB10878
SAMEA3541526	Control	PRJEB10878
SAMEA3541527	Control	PRJEB10878
SAMEA3541528	Control	PRJEB10878
SAMEA3541529	Control	PRJEB10878
SAMEA3541530	Control	PRJEB10878
SAMEA3541592	Control	PRJEB10878
SAMEA3541593	Control	PRJEB10878
SAMEA3541477	Control	PRJEB10878
SAMEA3541479	Control	PRJEB10878
SAMEA3541489	Control	PRJEB10878
SAMEA3541493	Control	PRJEB10878
SAMN00829166	Control	PRJNA46321
SAMN00829167	Control	PRJNA46321
SAMN00829168	Control	PRJNA46321
SAMN00829169	Control	PRJNA46321
SAMN00829170	Control	PRJNA46321
SAMN00829173	Control	PRJNA46321
SAMN00829174	Control	PRJNA46321
SAMN00829177	Control	PRJNA46321

References

1. Coker OO, Wu WKK, Wong SH, Sung JJ, Yu J. 2020. Altered gut archaea composition and interaction with bacteria are associated with colorectal cancer. *Gastroenterology* 159:1459-1470. e5.
